# Open collaborative smart plugs for energy management

**DOI:** 10.1016/j.ohx.2024.e00549

**Published:** 2024-06-24

**Authors:** Almir Neto, Luis Gomes, Zita Vale

**Affiliations:** a Federal Institute of Education, Science and Technology of Maranhão (IFMA), Av. Getúlio Vargas, n 04, Monte Castelo, São Luís, MA, 65030-005, Brazil; b GECAD-Research Group on Intelligent Engineering and Computing for Advanced Innovation and Development, Polytechnic of Porto (P.PORTO), P-4200-465 Porto, Portugal

**Keywords:** Energy analyzer, Internet of Things, Machine Learning, Smart plugs, Edge Impulse

## Abstract

Given the growth of domotics and home automation, there is a need to use smart devices that integrate energy management systems and enable the automation of the environment. Considering the need to study the relationship between the environmental parameters in which the equipment is located and the energy parameters, an Environmental Awareness smart Plug (EnAPlug) is proposed with the application of machine learning (Tiny ML).This article presents a demonstration of EnAPlug applied to a refrigerator for predictions on internal humidity and activation motor for 5 min-ahead prediction on its operation, i.e., turning on or off. The two models for forecasting humidity presented Root Mean Squared Error (RMSE) results of 0.055 and 0.058 and a Coefficient of determination (r2 score) of 0.97 and 0.99, respectively. For the motor activation prediction, the results obtained were an accuracy of 94.74% and 94.84%, an F1 score of 0.97 for OFF, 0.94 for ON for Forecast 1 and 0.97 for OFF and 0.93 for ON for Forecast 2. Although the prototype does not have commercial purposes, what differs from existing smart plugs is the option to store data locally. The results are promising, as it allows for better energy management with implementation of machine learning.

## Graphical abstract

**Figure:**
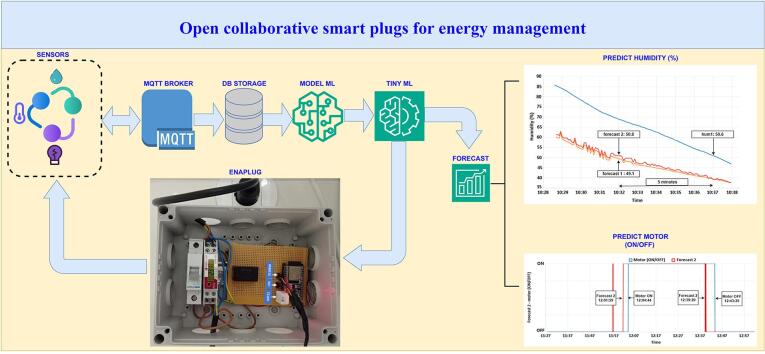

Specifications table

**Table:** 

Hardware name	*ENAPLUG (Environmental Awareness smart Plug)*
Subject area	*Engineering* *Internet of Things*
Hardware type	*Measuring physical properties and in-lab sensors*
Open source license	*Creative Commons Attribution-ShareAlike license.*
Cost of hardware	*162.24 EUR*
Source file repository	*https://doi.org/10.5281/zenodo.10825833*

## 1 Hardware in context

In view of the growth of home automation, there is a need to use smart devices that integrate energy management systems and enable the automation of the environment. In the market, there are a wide variety of smart plug models, however, one of the great difficulties encountered is the access to measurement data, limitation of energy parameters, application with other sensors and actuators, and the absence of machine learning models in the devices. Currently, smart plugs are intended to collect parameters from the power grid, such as current, voltage, power, energy consumption and others, but other parameters such as temperature, humidity, luminosity can directly influence energy consumption.

Given the need for conservation, the reduction of energy costs and the environmental impacts, the use of technologies that assist in energy management becomes a great ally for the efficient use of energy. In [1] the impact of using smart home technologies on energy conservation and demand management is studied. The study highlights the use of sensors, actuators and advanced systems in smart homes as a way to contribute to energy savings. The research results highlight an average reduction of 30 % in energy consumption and a reduction of up to 20 % in peak demand resulting in energy efficiency.

A study carried out in [2] highlights the need of users for intelligent solutions to reduce energy consumption and that in the United States, at least 30 % of the energy consumed by commercial and residential consumers is wasted, in addition, it presents a smart home system that aims to reduce electricity consumption by providing users perspectives regarding their consumption habits.

Home automation combined with big data, machine learning and the internet of things are great allies for energy efficiency [3]. In [3] a proposal that uses the Internet of Things (IoT) and Machine Learning that enables local or remote access by users is addressed. The article proposes the development of an intelligent mobile application (IntelihOgarT) that detects user behavior patterns and through this information recommends some options that provide comfort to users and recommendations that help save energy.

Similar studies presented indications of the use of platforms with the application of Internet of Things (IoT) and Machine Learning. As a contribution, EnAPlug proposes applications with embedded edge devices using IoT and Machine Learning for predictions that can contribute to energy consumption, in addition to the possibility of control, management of smart devices, access to data from sensors and actuators and data from parameters of the environment in which the device is located, such as temperature, luminosity and others. As a way to propose better energy efficiency.

According to the context, the scientific contribution of this proposal lies not only in the inclusion of machine learning in edge devices, but also in the contribution of analysing environmental parameters in which the device is located and which directly interfere with energy consumption, i.e., not only analysing power, current, power factor, reactive power, but also environmental factors such as temperature, humidity, luminosity in which the equipment is inserted.

The first version of EnaPlug was proposed in 2017, with application in a refrigerator, according to reference [4], containing an Arduino Mega, an Ethernet Shield, a MAX 485, a power analyzer (CVM-1D), a door opening sensor, external and internal temperature sensors, an internal humidity sensor, and a relay. References [4], [5], [6], [7] highlight the use of the Environmental Awareness smart Plug (EnAPlug) as a solution for application in a research and study centers to enable the study, testing, and validation of methodologies and models for energy management inside buildings. In [6], a new update is proposed that allows access to the operating system, enabling the processing and storage of data with a focus on the possibilities of learning in the consumption profiles and habits of users, in addition to the sharing of information between the different EnAPlugs. In article [7] EnAPlug is used for the integration of artificial intelligence with the acquisition of sufficient data for energy management or for decision-making through devices, through the application of Multi-Agent System (MAS).

EnAPlug’s proposal consists of integrating several sensors to better understand the environment where the specific load is located and not just limited to energy consumption, operating status, activation, and control [6]. EnAPlug, compared to Smart plugs available on the market, stands out for exploring information about the environment in which the device is inserted, such as temperature, humidity, luminosity and other important parameters that can be entered through new sensors, i.e., data that can directly or indirectly affect the results of energy management, in addition, it allows access to data locally, differentiating it from others that are done centrally and in the cloud, in addition to enabling the application of machine learning in embedded systems and edge, through Tiny Machine Learning (TinyML) [5], [6].

It is important to clarify that EnAPlug is intended for teaching purposes and for research centres and has no commercial purposes, nor is it intended to replace technologies already used in refrigerators with inverter or intelligent refrigerator technologies, but rather to contribute with analyses and possible implementations, such as humidity forecasts for better food preservation and motor activation to reduce energy consumption.

One of the great contributions of hardware is the implementation of machine learning on edge devices (Tiny Machine Learning). Another contribution is the analysis of environmental parameters in which the device is inserted associated with energy parameters to evaluate the equipment's consumption characteristics.

The use of EnAPlug can be used as a complement to the analysis of reducing energy costs using other parameters that influence energy consumption. For instance, we can mention the energy savings that can be obtained from data such as water heater temperature, associated with energy data, such as active power and current to predict motor activation and thus prevent it from being activated during peak hours.

As a form of validation, two case studies were proposed, one for humidity and the other for motor activation. For each test, two neural network models were developed with the aim of evaluating the best performance, being “classification” for predicting motor activation and “regression” for predicting humidity.

**Fig. 1:**
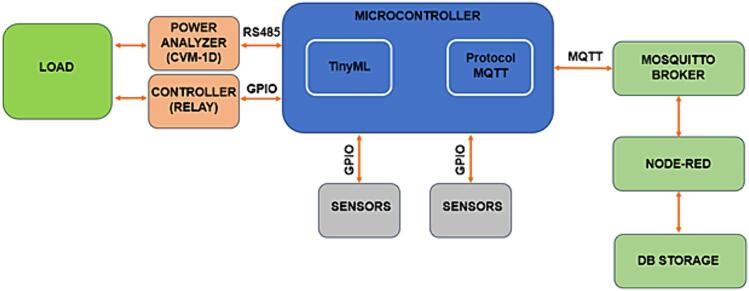
Architecture proposed of EnAPlug.

This article proposes the presentation of the EnAPlug hardware with the aim of motivating and stimulating the development of new models and versions for energy management applications and contributing to the improvements of EnAPlug, as this is not a commercial product [7]. Fig. 1 shows the architecture proposed of EnAPlug [4], where:

• LOAD – the LOAD can be any equipment to be monitored or controlled by EnAPlug. In this project, a refrigerator was used as LOAD;
• POWER ANALYZER – this module reads energy parameters. For this project, the CVM-1D power analyzer was used, which measures current, voltage, active power and reactive power. It uses RS485 serial communication, through the Modbus/RTU protocol, to send and receive data between the microcontroller and the MAX485;
• CONTROLLER – It is used to turn the load on and off, such as using a relay;
• MICROCONTROLLER – It is responsible for all information processing, sensor readings and actuators activation, in addition to reading and sending data to the Mosquitto Broker, through the MQTT protocol. One of its main advantages is the possibilities of applying machine learning, through TINYML (Tiny Machine Learning), enabling the integration of artificial intelligence in edge devices, in addition to low energy consumption and application of the MQTT protocol. The microcontroller used was the ESP32 which is dual-core, has WiFi, Bluetooth and peripherals for RS-485 communication and General Purpose Input/Output (GPIO);
• MOSQUITTO BROKER – through the MQTT protocol, the microcontroller and sensors send and receive messages, in JSON format, through the use of Publish-Subscribe;
• NODE-RED − for control and integration between sensors, actuators and microcontroller, the Node-RED platform was used, which is a tool that facilitates the application of the Internet of Things (IoT). For this project, Node-red receives data through topics and displays it through a dashboard, in addition, it sends the data for storage in DB STORAGE.
• DB STORAGE – it is used to store sensor data and through this data enable the training of the neural network to create the machine learning model.

After this section, section 2 presents hardware descriptions highlighting the main components and their applications in the project. 3, 4 list the files and components used to develop the project. Instructions for assembling the circuit and presenting EnAPlug are described in section 5. The steps for operating instructions, with descriptions of software installation and hardware implementation, are covered in section 6. Finally, in section 7, the validation, operation and performance of EnAPlug with the application of machine learning and the results obtained are presented.

## 2 Hardware description

EnAPlug has an open interface that allows integration with various models of sensors and actuators. The proposed EnAPlug has the ESP32 [9] microcontroller, produced by Espressif [10], which facilitates the implementation of machine learning, using TinyML, and the Message Queuing Telemetry Transport (MQTT) protocol [11]. The specifications of the ESP32 are described in Table 1 [9].

**Table 1:** Specifications of ESP32.

Microprocessor unit	ESP32 (240 MHz Tensilica dual-core 32 bits, 520 KB Static random-access memory (SRAM), Wi-Fi (802.11b/g/n), Bluetooth v4.2 Bluetooth basic rate/enhanced data rate (BR/EDR) and Bluetooth Low Energy (BLE), 4 MB External Serial Peripheral Interface (SPI) FLASH)
Integrated crysta	40 MHz crystal
Communications Protocols	Universal Asynchronous Receiver/Transmitter (UART); Serial Peripheral Interface (SPI); Inter-Integrated Circuit (I2C); Inter-IC Sound (I2S)
FLASH	4 MB Serial Peripheral Interface (SPI) FLASH

The power analyzer (CVM-1D) has a Liquid Crystal Display (LCD) display, that allows the reading of 25 variables instantly (maximum and minimum values) and uses the Modbus/ Remote Terminal Unit (RTU) (RS-485) protocol for communication, the information is described in Table 2 [14].

**Table 2:** Power analyzer (CVM-1D).

Consumption	2VA
Frequency	50/60 Hz
Nominal voltage	110…230 Vac ± 20 %
Nominal current (In)	5 A (min./max.: 0.250…32 A)
Reference current (Iref)	5 A
Communications	RS-485
Protocol	Modbus/RTU

The Temperature and Humidity Sensor(DHT22) [21] sensor was used to measure temperature and humidity [22], which is a digital sensor [23] that reads temperature with a variation from −40 °C to 80 °C ± 0.5 °C and air humidity from 0 to 100 % ±2-5 % [24], as described in Table 3.

**Table 3:** DHT22.

Power supply	3.3 – 5 VDC
Output signal	1-wire bus digital signal
Operating range (humidity)	0 – 100 % RH
Operating range (temperature)	*−*40 *∼* 80 *^◦^C*
Accuracy (temperature)	*±*0*.*5 *^◦^C*
Accuracy (humidity)	±2 −5 % RH
Protocol	Modbus/RTU

For RS-485 serial communication [12] between the power analyzer (CVM-1D) and the microcontroller, the MAX485 serial interface was used, which is a low-power transceiver for RS-485 [13]. The microcontroller reads data from the power analyzer, using the MODBUS/RTU protocol [6], and sends it to the MQTT BROKER [7]. EnAPlug sends and receives data, through Publish and Subscribe, using the MQTT protocol [8]. Installing MQTT BROKER to apply the MQTT protocol is possible by installing Eclise Mosquitto Broker [28].

Mosquitto Broker is an open-source message broker used in Internet of Things (IoT) applications for communication between devices [28]. The MQTT protocol is used for communication between devices, sensors, actuators, M2M (Machine to Machine) communication widely used in Internet of Things (IoT) applications, through publish/subscribe, where it sends and receives data from sensors that are registered in specific topics [28]. In this project, the MQTT protocol was used to send and receive data from the refrigerator, such as: voltage, current, active power, temperature and humidity.

The other sensor used to measure the temperature was the DS18B20 sensor, as it allows measurement in humid or wet locations and its specifications are described in Table 4 [31].

**Table 4:** DS18B20.

Power supply	3.0 – 5.5 VDC
Output signal	1-wire bus digital signal
Measures Temperatures	*−55 °C to + 125 °C*
Accuracy	*±*0*.*5 *^◦^C*
Programmable Resolution	9 to 12 bits

Node-Red, used in this work as centralized platform, is a free, open source, flow-based programming, low-code platform used for Internet of Things (IoT) applications [25] and is developed using the Node.js programming language [8]. Nodes can be easily dragged and connected to each other, in addition, it has integration with several other services and consists of the following components: Node menu, Flow panel, Info and Debug menu, in addition, it allows you to export and import flows [25], install new nodes and enables the implementation of various protocols, such as MQTT [26]. Once installed locally, access to the platform can be done via the address: https://127.0.0.1:1880/ [25].

Neural networks are typically trained and executed using large computational power [15]. In the context of Artificial Intelligence for Edge machine learning (EDGE ML) in which data is processed, stored and managed at the edges, the ESP32 are good options for implementing machine learning (ML), through TinyML [16]. TinyML makes it possible to implement ML on edge microcontrollers, with low latency, low bandwidth, privacy and low energy consumption, providing autonomy in decision making. The data read by the sensor is processed on the device itself without the need to send it to cloud processing [17].

Edge Impulse is an artificial intelligence (AI) application platform that enables the development and deployment [15] of ML embedded devices at the edge [20]. According to [16], Edge Impulse is a cloud-based machine learning operations (MLOps) platform for applying embedded and edge systems. It makes it possible to simplify data collection, training neural networks and embedding them on edge devices [16]. The use of Edge Impulse provides the use of ML in low-power systems, with limited resources, using TinyML and has enabled the application of several projects in systems entangled at the edges [19]. Furthermore, it allows the use of the C++ library, Arduino library and WebAssembly, which will be used in this project [18]. The proposed hardware has the following contributions:

• Allows you to explore information about the environment the device is inserted, such as temperature, humidity, brightness and other important parameters;
• Enables access to data locally or in the cloud, differentiating it from others that are done centrally and in the cloud;
• Enables the reading of 25 energy parameters through the CVM-1D power analyzer, such as: Voltage, Current, Active Power, Reactive Power, Apparent Power, Maximum demand, Power Factor, Active Energy, Reactive Energy, Partial Active Energy, Partial Reactive Energy and others [14];
• Allows the application of Machine Learning in embedded and edge systems through TinyML.

## 3 Design files summary

The design files summary are described in the Table 5.

**Table 5:** Design files.

**Design filename**	**File type**	**Open source license**	**Location of the file**
Architecture proposed of EnAPlug	.png	GNU General Public License (GPL) 3.0	https://doi.org/10.5281/zenodo.10825833
Circuit of EnAPlug	.png	GNU General Public License (GPL) 3.0	https://doi.org/10.5281/zenodo.10825833
Flows.json	.js	GNU General Public License (GPL) 3.0	https://doi.org/10.5281/zenodo.10825833
Enaplug.ino	.ino	GNU General Public License (GPL) 3.0	https://doi.org/10.5281/zenodo.10825833
Enaplug.sql	.sql	GNU General Public License (GPL) 3.0	https://doi.org/10.5281/zenodo.10825833

## 4 Bill of materials summary

The list of components required for the EnAPlug is described in Table 6.

**Table 6:** Bill of materials.

**Designator**	**Component**	**Number of units**	**Cost per unit − EUR**	**Total cost − EUR**	**Source of materials**	**Material type**
ESP32	Microcontroller (Espressif)	1	12.50	12.50	https://www.botnroll.com	Electronics
DHT22	Digital-output relative humidity & temperature sensor	1	11.60	11.60	https://www.botnroll.com	Electronics
Resistor	Resistor 10KW	1	0.05	0.05	https://www.botnroll.com	Electronics
DS18B20	Digital Thermometer sensor	1	3.57	3.57	https://mauser.pt	Electronics
MAX485	Low-power transceivers for RS-485 and RS-422 communication	1	5.40	5.40	https://mauser.pt	Electronics
CVM-1D	Power Analyzer	1	142.42	142.42	https://solarmat.es	Electronics
HAGER MW 116 C16	Protection Devices	1	6.94	6.94	https://hager.com	Electronics
HLK-PM01 230VAC to 5 V/3W DC	Ultra-compact Power Module	1	3.4	3.4	https://mauser.pt	Electronics
LDR	Light Dependent Resistance	1	0.30	0.30	https://mauser.pt	Electronics

## 5 Build instructions

**Fig. 2:**
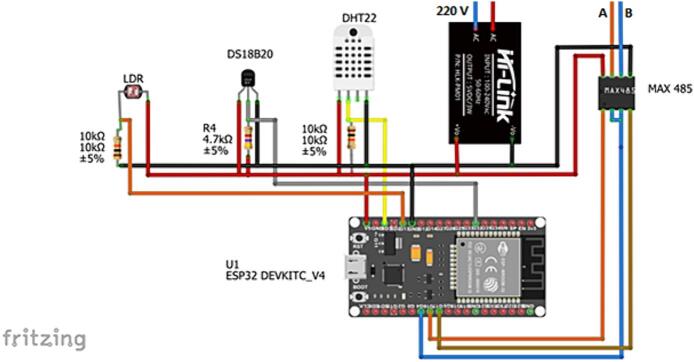
Circuit of EnAPlug.

Fig. 2 shows the EnAPlug circuit with the sensor connections to the microcontroller, in which the temperature and humidity sensor (DHT22) is connected to GPIO pin 10 (digital), as the DHT has an internal analog-to-digital converter that facilitates sensor reading and communication with the microcontroller. The Light Dependent Resistor (LDR) is connected to pin 13 of the microcontroller that has the ADC converter and through the “analogRead” function the analog reading of the sensor is made. The MAX485 converter allows RS485 serial communication between the microcontroller and the power analyzer. The Drive Enable Input (DE) and Receiver Enable Input (RE) pins are connected to GPIO 4, the Receiver Output (RO) pin is connected to pin 16 (UART RX) and the Drive Input (DI) to pin 17 (UART TX) and pins A and B are connected to the power analyzer. The DS18B20 sensor is used for temperature measurement and the data pin is connected to pin 32, VCC to 5v and GND to GND. The sensors were not fixed directly to the board, but inputs were made available for connecting the sensors, as this facilitates the application.

**Fig. 3:**
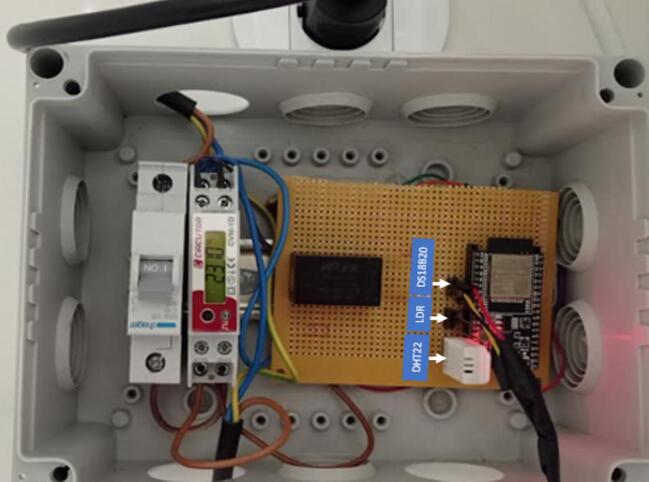
EnAPlug.

After assembling the circuit, it is necessary to connect the flexible cable (3 x 1.5 mm^2^) to the male and female plugs and then connect them to the circuit breaker and power analyzer (CVM-1D). Do not carry out these procedures with the circuit switched on, as they may pose risks. Fig. 3 shows EnAplug.

## 6 Operation instructions

After assembling the hardware, it is necessary to install Arduino IDE that is described in [29]. The next step is integrated and control devices through topics. The Node-Red platform is used to facilitate the reading and visualization of sensor data and actuator control through the MQTT protocol. The “flows.json” file has the configuration used and its installation is described in [27].

To send and receive the data through the MQTT protocol, the Mosquitto Broker was used and its installation is described in [28]. To use the database, the XAMPP software was used and the installation is described in [30]. Once the installations are done, it is necessary to activate MQTT Broker (mosquitto –v) and start the “Apache” and “MySQL” modules in XAMPP. After, execute the commands described in the “enaplug.sql” file. Data storage is done through the “mysql” node and its installation is done through “node-red-node-mysql” on Node-Red.

The next step is to open the.ino file (Enaplug.ino) and change the Wi-Fi network settings and the MQTT Broker server address. Once the changes have been made, it is necessary to turn on the microcontroller and check whether the connection with the Broker, NODE-RED and the Database have been established, if so, check whether the data is being stored correctly, otherwise repeat the previous steps and check if anything is missing.

After that, it is necessary to train the neural network and for this, we used the Edge Impulse platform. Access to the Edge Impulse website is via the address: https://edgeimpulse.com/ [20]. First you need to register and then access the project available at the address: https://studio.edgeimpulse.com/.

After accessing the platform, it was necessary to include the dataset with sensor data in “Data acquisition”, then the parameters were defined in “Impulse Design”. In the “Create impulse” option, the “Time series data” fields were configured to 1 Hz, in “Add a processing block” the “Raw Data” option was chosen for data without pre-processing. In “input axes” the inputs collected from the dataset are identified (act1, temp2, hum1, curr1), in the option “Add a learning block” “Regression” was chosen for the humidity prediction, and “Classification” for the motor activation prediction. The settings of each model are described in Table 9, Table 10. Once the results were satisfactory, the next step was to validate the model in “Model testing” and finally, “Deployment” was carried out to implement the trained model on the microcontroller (WebAssembly or Arduino library). For testing purposes, “WebAssembly”, installed on NODE-RED through the “edge-impulse-classify” node, and the “Arduino library” were used. Finally, once the model has been implemented in the microcontroller, it is possible to compare the actual values ​​with the predicted values.

**Table 7:** Inputs and output to predict motor ON/OFF (forecast 1 and forecast 2).

Inputs (forecast 1 − motor)	Output
Temperature (°C) – temp2	Motor ON / OFF
Active power (W) – act1	
Current – curr1	
Humidity (%) – hum1	
Inputs (forecast 2 − motor)	Output
Temperature (°C) – temp2	Motor ON / OFF
Active power (W) – act1	
Humidity (%) – hum1

**Table 8:** Inputs and output to predict humidity indoor (forecast 1 and forecast 2).

Inputs (forecast 1 − humidity)	Output
Temperature (°C) – temp2	Humidity indoor (refrigerator)
Active power (W) – act1	
Current – curr1	
Humidity (%) – hum1	
Inputs (forecast 2 − humidity)	Output
Temperature (°C) – temp2	Humidity indoor (refrigerator)
Active power (W) – act1	
Humidity (%) – hum1

**Table 9:** Forecast 1 and forecast 2 to predict Motor ON/OFF.

**Validation**	**Forecast 1 − motor**	**Forecast 2 – motor**
Train Accuracy (%)	95.6	95.1
Test Accuracy (%)	94.74	94.84
Batch size	32	32
Optimizer	Adam	Adam
Activation	Relu	Relu
Epochs	30	30
Learning rate	0.0005	0.0005
Dense layer at first level (neurons)	20	20
Dense layer at second level (neurons)	10	10
Input layer forecast 1 − motor Input layer forecast 2 − motor	4 (curr1, temp2, hum1, act1)	3 (temp2, hum1, act1)
Output layer (ON/OFF)	2 (ON/OFF)	2 (ON/OFF)
Inferencing time (ms)	1	1
Peak RAM Usage (bytes)	1.4 K	1.4 K
Flash Usage (bytes)	14.9 K	14.8 K

**Table 10:** Forecast 1 and forecast 2 to predict humidity indoor (refrigerator).

**Validation**	**Forecast 1 − humidity**	**Forecast 2 − humidity**
Accuracy (%)	96.42	98.66
Batch size	32	32
Optimizer	Adam	Adam
Activation	Relu	Relu
Root Mean Squared Error − RMSE	0.055	0.058
Coefficient of determination (r^2^ score)	0.97	0.96
Epochs	200	100
Learning rate	0.005	0.005
Dense layer at first level (neurons)	150	20
Dense layer at second level (neurons)	50	10
Input layer forecast 1 − humidity Input layer forecast 2 − humidity	4 (curr1, temp2, hum1, act1)	3 (temp2, hum1, act1)
Output layer (humidity indoor)	1	1
Peak RAM Usage (bytes)	1.4 K	1.2 K
Flash Usage (bytes)	18.9 K	10.5 K

## 7 Validation and characterization

In the project validation process, the refrigerator was previously monitored and the data from the sensors were stored in a database and soon after the data was implemented for training the neural network. The research group has a database with one year's worth of refrigerator data, but for the tests, only one month's data was analysed, being chosen the month in which the greatest intervention occurred in the refrigerator.

The proposal is intended for the application of motor activation forecast and the internal humidity of the refrigerator, however, this application is not limited to this, taking into account the possibilities of other applications, such as energy savings generated by the activation forecast of a water heater motor or controlling the air conditioning temperature forecast during peak hours to reduce energy consumption.

In order to validate the proposed project, data was read for approximately one hour. The tests were carried out on only one refrigerator model, but they can be expanded for a greater comparison of data between refrigerators.

During the test period, the refrigerator door was not opened, as opening the door influences the prediction of internal humidity, as it cannot be predicted when the user will open the door, however, according to the database, the average time the refrigerator door was opened was approximately 5 s and the total number of times the door was opened was greater than 300 times during the month. Once the door is opened, the humidity forecast follows the current humidity values ​​and after closing it returns to the predicted data. For motor prediction, opening the door does not have the same effect as humidity, however the time the door is opened, the number of times it is opened, the temperature and internal humidity will influence the motor activation.

This project aims to analyse the parameters that might influence energy consumption. Since it is not possible to carry out interventions in the refrigerator due to food conservation, such as turning it off during peak hours, for example, forecast data can be useful for better management of the use of renewable energy sources during busy times peak.

The relevance of using EnAPlug lies in the use of other parameters of the environment in which the device is located, such as temperature, humidity, and luminosity, not just electrical energy data. To validate the prototype, two tests are proposed, the first using the temperature, humidity, current, and active power data as a way of identifying whether the motor of a refrigerator will be on or off in the next 5 min and the second test to predict the internal humidity of a refrigerator in the next 5 min. For each test, two predictions (forecast 1 and forecast 2 for predict motor ON/OFF and forecast 1 and forecast 2 for predict humidity indoor) were made with different inputs, as shown in Table 7, Table 8.

**Fig. 4:**
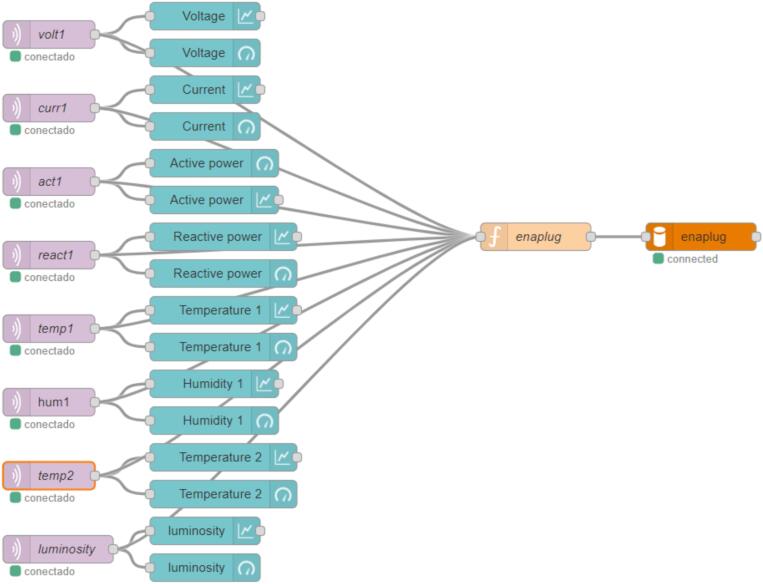
Flows.

The readings made by the microcontroller (ESP32) are sent via the MQTT protocol to the MQTT Broker and stored in the database. Data is received, by Node-Red, through the topics: voltage (volt1), current (curr1), active power (act1), reactive power (react1), Light Dependent Resistor (luminosity), temperature DHT22 (temp2), humidity DHT22 (hum1) and temperature DS18B20 (temp1). The values ​​read through the topics are displayed through the “gauge” and “chart” dashboards. An “enaplug” function is responsible for collecting information from the sensors, such as the name of the topic “name”, read values “value”, the date and time “timestamp”. The “mysql” node, identified as “enaplug”, allows access to the MySQL database and through this, the data is stored, as shown in Fig. 4.

**Fig. 5:**
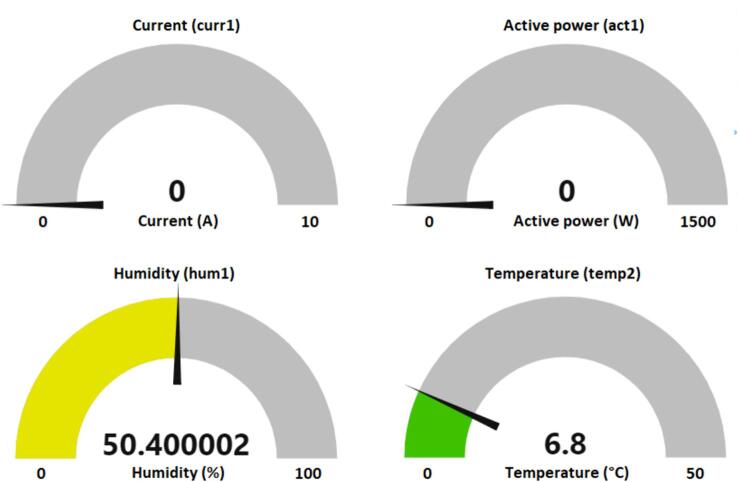
Dashboard gauge.

In addition, the collected data is displayed on the dashboard. Fig. 5 shows “dashboard gauge” with the values of temperature DHT22 (temp2), humidity DHT22 (hum1), current (curr1) and active power (act1). The values indicated in Fig. 5 represent the moment when the motor is off with active power and current values equal to zero. The internal humidity of the refrigerator is 50.40 % and the internal temperature is 6.8 °C.

**Fig. 6:**
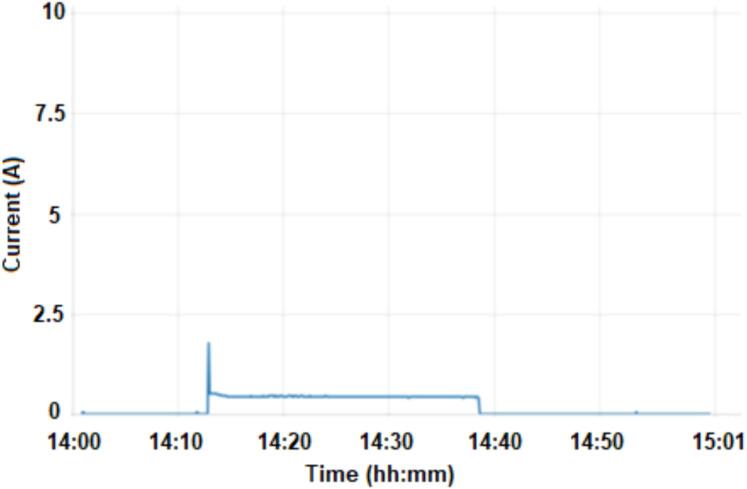
Dashboard chart current (curr1).

**Fig. 7:**
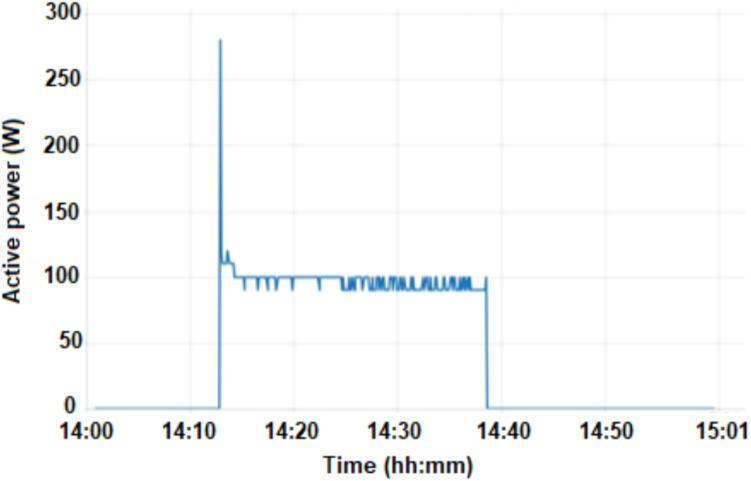
Dashboard chart active power (act1).

**Fig. 8:**
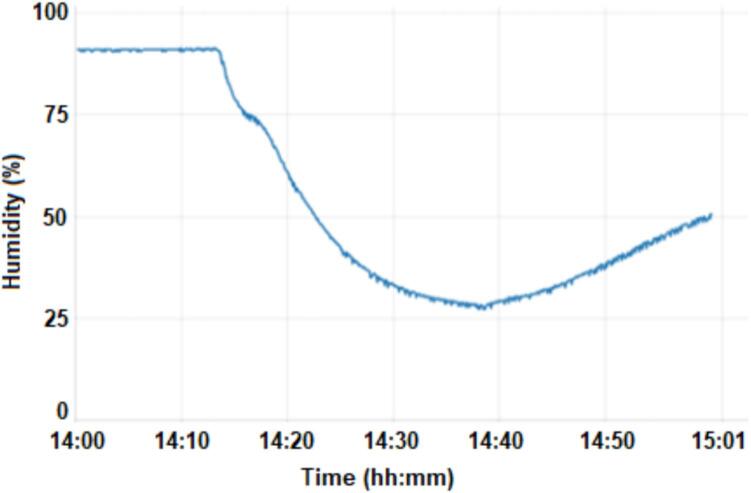
Dashboard chart humidity (hum1).

**Fig. 9:**
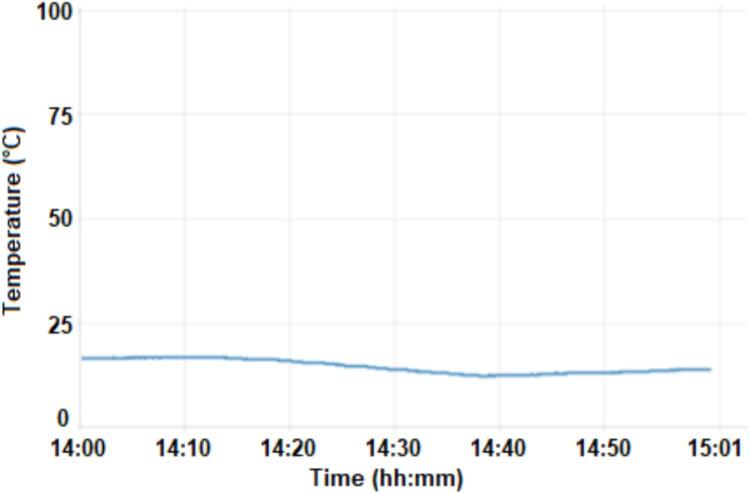
Dashboard chart temperature (temp2).

Fig. 6, Fig. 7, Fig. 8, Fig. 9. shows the “dashboard chart” with input data in function of the time and the values of current (curr1), active power (act1), humidity DHT22 (hum1) and temperature DHT22 (temp2). Fig. 6 shows the current values (A) for one hour. A peak value of approximately 1.88 A is detected at the start of the motor, at 14:13, this value refers to the “start” of the motor. The average current value during refrigerator operation is 450 mA, indicated in the figure, from 14:13 to 14:38, approximately. The average door opening time, according to the dataset, is approximately equal to 5 s. The first period in which the refrigerator is on lasts approximately 25 min (2:13 pm to 3:38 pm).

Fig. 7 shows the Active Power graph, with the activation period being similar to the current. In the first instant of motor operation, there is a peak power at motor start-up of approximately 280 W at 14:13. During the period of motor operation the average power of the refrigerator is approximately 100 W. When the refrigerator door is opened, the 20 W internal lamp is turned on, generating a current of approximately 110 mA.

According to Fig. 8, it is observed that there is a decrease in humidity during the period in which the motor is running, that is, it reduces from 90 % when the motor starts, to 26 % when the motor turns off, corresponding to a variation of 64 %. According to the dataset, if we compare the activation time and the change in the humidity value, it can be seen that the humidity varies 35 s after the motor started and 15 s after the motor turned off.

Temperature measurements are shown in Fig. 9. The average temperature during the measurement period was approximately 8 °C. When the motor is running, the temperature drops by approximately 1 °C, that is, an average motor operating time of 25 min. Humidity and temperature have an immediate response when the door is opened, due to the exchange with the environment, and the time for recovery of initial values ​​is related to the time it remained open. This data is important, as it impacts the time the motor will run until it reaches standard values.

In this project, the Edge Impulse was used to train and validate the machine learning [19]. The quality and type of data collected at this stage (data acquisition) are important, as they result in better network training. The data collected in real time is sent through the MQTT protocol, through topics, and stored in the database in (.csv) format. In EDGE Input, the database is sent through the “CSV Wizard” option and in “Dataset” the data is separated into a percentage of 20 % for testing and 80 % for training.

Next, the input and output data, hyperparameters, learning block and Neural Network settings [18] are configured. The model used to predict the motor (ON/OFF) was “Classification” in which the model output indicates whether the motor will turn on (ON − 1) or whether it will turn off (OFF − 0). The algorithms used for motor ON/OFF prediction, Forecast 1 and Forecast 2, have the following characteristics, the number of training cycles equal to 30, that is, the number of epochs to train the neural network, a learning rate of 0.0005, the size of the data validation set is 20 %, the batch size used during training is 32, optimizer “Adam” and the activation function “relu”. Forecast 1 has 4 input variables (curr1, temp2, hum1, act1) and Forecast 2 has 3 input variables (temp2, hum1, act1). Forecast 1 and Forecast 2 have two dense layers with 20 and 10 neurons, respectively, and two output layers (ON/OFF).

To predict Motor ON/OFF, the EDGE IMPULSE “Classifier” classification model was used. After the training data, the model presented good results with an accuracy of 95.6 % (forecast 1) and 95.1 % (forecast 2) for testing results an accuracy of 94.74 % and 94.844. The forecast parameters and results (forecast 1 and forecast 2) are presented in Table 9.

To predict the refrigerator's indoor humidity, the model used was “Regression”, as it facilitates the prediction of continuous values and the output will indicate the predicted values ​​for humidity. The parameters used for the humidity prediction neural network were a batch size of 32, learning rate of 0.005, “Adam” optimizer and the “relu” activation function. Forecast 1 used 200 epochs, dense layer at first level with 150 neurons, dense layer at second level with 50 neurons, 4 input variables (curr1, temp2, hum1, act1) and one output (indoor humidity). For Forecast 2, 100 epochs were used, dense layer at first level with 20 neurons, dense layer at second level with 10 neurons, 3 input variables (temp2, hum1, act1) and one output (indoor humidity).

The parameters were selected according to the initial configuration indicated in Edge Impulse, and these were changed or maintained according to the model results. The results of the forecast 1 and forecast 2 (humidity) predictions showed good results with an accuracy of 96.42 and 98.66, respectively, and a Coefficient of determination (r2 score) of 0.97 and 0.99, as shown in Table 10.

**Fig. 10:**
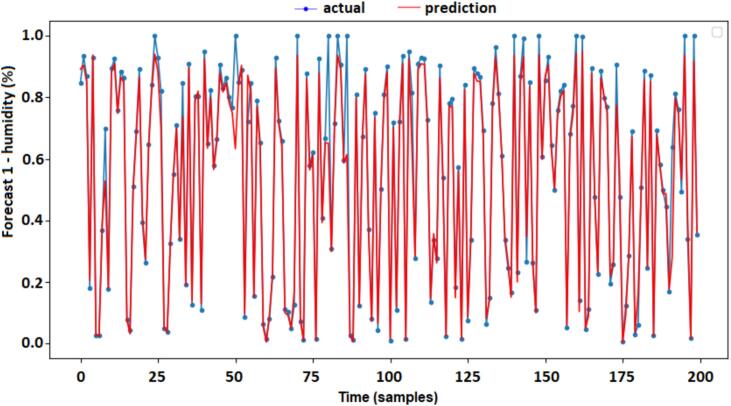
Actual vs predictions forecast 1 (humidity indoor).

An approximation of the prediction values and the actual values are shown in Fig. 10, this demonstrates a good response of the Forecast 1 model in terms of predicting internal humidity (refrigerator) for the next 5 min. The range used to validate the tests was 200 samples. The simulation curves between the “actual” and “prediction” of forecast 1 (humidity indoor) are shown in Fig. 10.

**Fig. 11:**
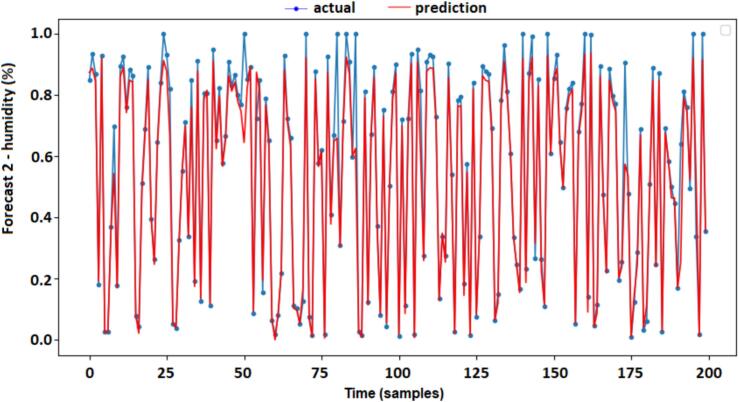
Actual vs predictions forecast 2 (humidity indoor).

A similar answer was also obtained from the simulated results of Forecast 2, which show, through Fig. 11, very close values ​​between the measurements of the actual and prediction values. It is concluded that the two models (Forecast 1 and Forecast 2) were successful in the proposed training. The result of curves between the “actual” and “prediction” of forecast 2 (humidity indoor) and are shown in Fig. 11.

**Fig. 12:**
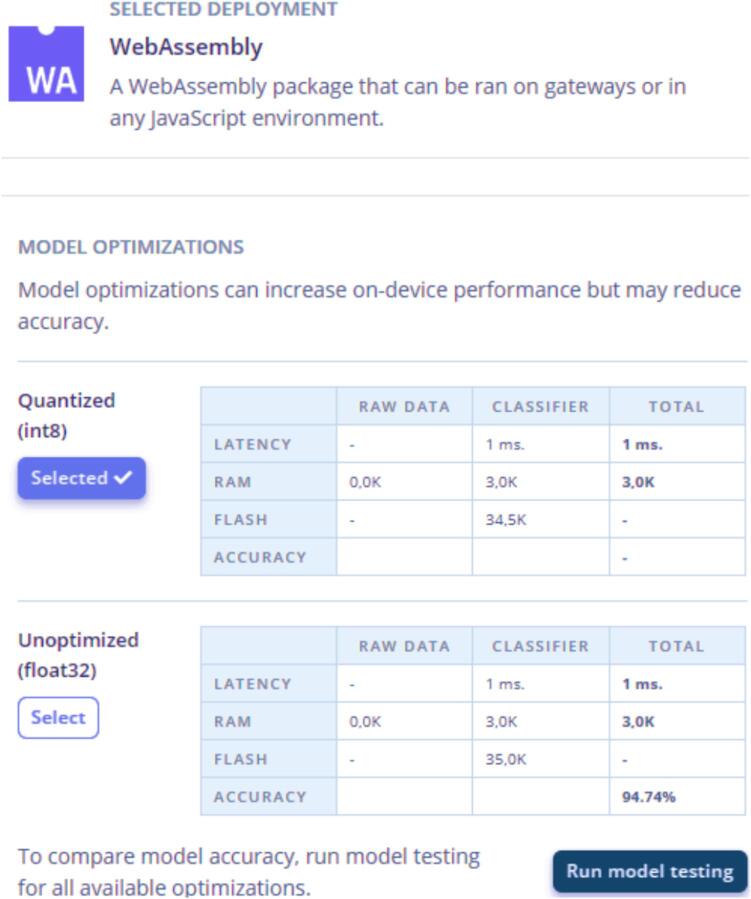
Deployment-WebAssembly.

The optimization models and deployment are identified in Fig. 12. Edge impulse offers a varied list of deployments, however in this project “WebAssembly” and the “Arduino library” were used as a deployment way of the model on the microcontroller and tests. Due to the memory limitations of the microcontroller, the quantized model (int8) was chosen, despite knowing about the losses related to the performance of the models. Final tests showed that the choice of quantized optimization (int8 bits) did not harm the intended results. The Edge Impulse makes it possible to embed the trained model on the microcontroller using different types of devices. In this project, the ESP32 microcontroller was used to embed the neural network and the Arduino and WebAssembly library to validate the trained model [14], as shown in Fig. 12.

After validation tests, the trained neural network was implemented in EnAPlug, according to the steps described at: https://docs.edgeimpulse.com/docs/run-inference/cpp-library/running-your-impulse-locally.

**Fig. 13:**
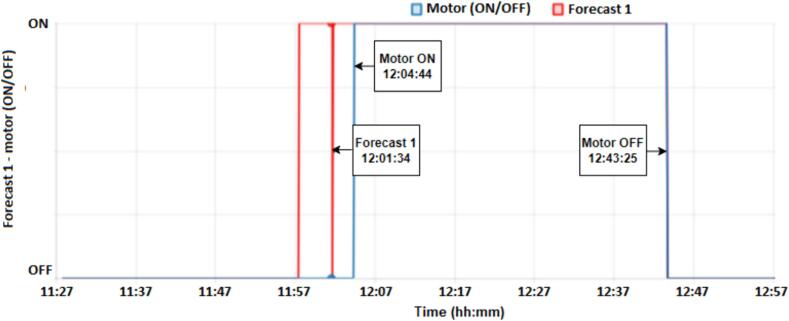
Result forecast 1 – motor (ON/OFF).

**Fig. 14:**
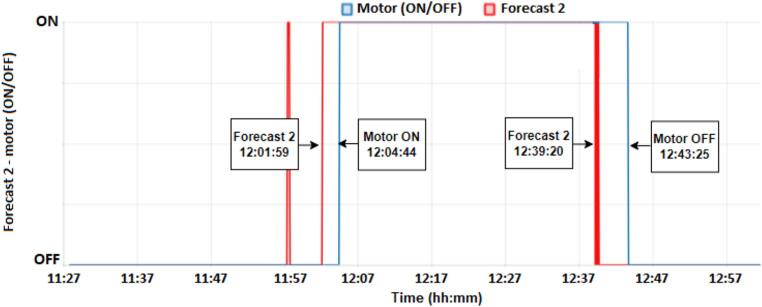
Result forecast 2 – motor (ON/OFF).

According to the results in Fig. 13 and Fig. 14, both Forecast 1 and Forecast 2 presented a correct prediction for activation (ON) with 03:10 and 02:45 before the motor starts, but only Forecast 2 managed to correctly identify the moment when the motor would shut down (OFF). As show to Fig. 13, the motor starts at 12:04:44 and turns off at 12:43:25, forecast 1 occurs at 12:01:34, that is, 03:10 before the motor starts (MOTOR ON), presenting a satisfactory result and within the expected time, however forecast 1 was unable to predict the moment when the motor would turn off (MOTOR OFF).

On the other hand, forecast 2 is activated at 12:01:59, that is, 02:45 before the motor is activated (MOTOR ON) and turns off at 12:39:20, that is, 04:05 before the motor is turned off. (MOTOR OFF), resulting in a better response to the proposed model, as shown in Fig. 14.

In all the tests, the predictions occurred moments before activation, that is, enough to indicate that the motor of refrigerator would turn on or off. In terms of application, it can be inferred by checking whether or not you want, for example, a water heater to turn on due to the schedule/tariff or just an indication.

Motor drive Forecast 1 presents a train accuracy of 95.6 %, test accuracy of 94.74 % and a loss of 0.14. In the model test results, the confusion matrix presents a result of 95.3 % accuracy for OFF (0), that is, accuracy when the response is OFF and 93.7 % accuracy for ON (1). The F1 score value is equal to 0.97 for OFF and 0.94 for ON. Forecast 2 has a train accuracy of 95.1 %, test accuracy of 94.84, and a loss of 0.17. In model testing output the confusion matrix shows a result of 96.5 % accuracy for OFF and 91.6 % for ON. The F1 score corresponds to 0.97 for OFF and 0.93 for ON. Although the F1 score, loss, and accuracy present similar values ​​in the two models, in practice, Forecast 2 presented a better response, identifying the two moments ON and OFF.

**Fig. 15:**
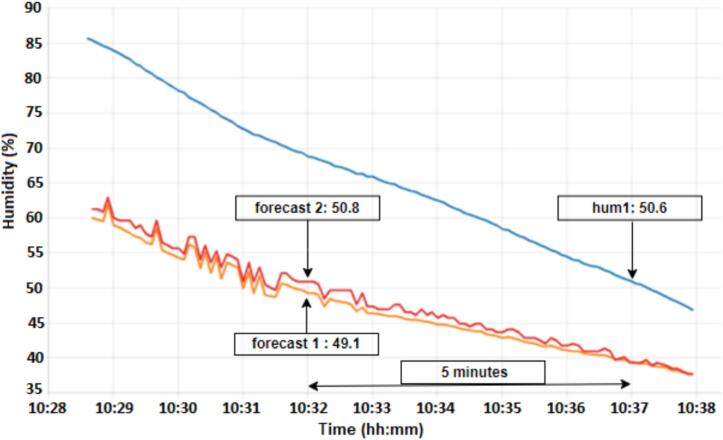
Results forecast 1 and forecast 2 (humidity).

From Fig. 15 it can be seen that the humidity forecast 1 and forecast 2 values ​​presented values ​​close to the actual forecast. According results in Fig. 15, the humidity forecast, follows the actual measured values and makes a forecast for the next few minutes. The forecasts (forecast 1 and forecast 2), light blue and orange colors, predict a value of approximately 97 % at the time 10:32:00 (forecast 1 in 49.1 and forecast 2 in 50.8) and at the time 10:37:00 the humidity (hum1 in 50.6), blue color, reaches the value predicted, that is, 5 min later, thus validating the proposed model and making it possible to control the humidity inside the refrigerator.

The two models for forecasting humidity (Forecast 1 and Forecast 2) showed accuracy results of 96.42 and 98.66, respectively. The Root Mean Squared Error (RMSE) values ​​were 0.055 and 0.058 and the Coefficient of determination (r2 score) was 0.97 and 0.96, respectively. According to the results, the values ​​present very close values, validating the two proposed models.

A highlight to be made for the two models (motor and humidity) is the influence of the input variables, as both were tested using 3 and 4 input variables. The model with 4 inputs for activating the motor did not obtain a good response for detecting the moment when the motor turns off (OFF). For humidity prediction, the two tests using 3 and 4 input variables showed a good response.

According to the results obtained, EnAPlug provides a varied application with the objective of analyzing the environment in which the device is inserted and, based on this information, predicts behaviors and characteristics that contribute to better electrical energy management. The difference between EnAPlug is the possibility of incorporating a trained neural network for better control of energy expenditure. EnAPlug has no commercial purposes and is intended for teaching purposes and research groups.

## 8 Conclusions

This article proposed open collaborative smart plugs for energy management that use the characteristics of the environment in which the equipment is inserted, such as humidity, and temperature, associated with energy parameters, such as power and current to analyse and make predictions that help in better energy management using machine learning on an edge device (TinyML).

Through a practical application, described in the validation and characterization section, it was possible to validate and test the prototype with predictions for the activation of an motor and the internal humidity of a refrigerator for the next few minutes.

The results demonstrated that the proposal was successful. The studies made it possible to identify relationships between the environmental parameters (temperature and humidity) in which the equipment is inserted and the energy data (current and active power), making it possible to make predictions that can contribute to reductions in energy consumption. In this application, a refrigerator was used, but the analysis can be extended to other equipment, such as air conditioning and water heater, where it is possible to predict motor activation and humidity and thus avoid activation during on-peak hours and enable better electrical energy management. Therefore, it is concluded that the proposed prototype contributes to studies and analyses related to energy efficiency, considering the low cost for assembling and installing the software, in addition, it is a collaborative system.

Although machine learning models present a good response, it is suggested to include more data and adjust the hyperparameters to obtain a better response for the proposed model. To better investigate the performance of the results, it is suggested to extend the test validation time. It is also suggested to investigate the energy consumption generated by the number of times the door is opened.

The novelty of the proposed prototype is the inclusion of machine learning in an embedded edge system with analysis of energy parameters and environmental parameters for energy management purposes.

According to the results obtained, the open collaborative smart plugs for energy management is a tool that contributes to analysis and research to evaluate behaviours based on parameters of the environment in which the equipment is located aiming better energy management.

The use of artificial intelligence embedded in an edge device using energy and environmental parameters is one of the project's contributions, as it contributes to the development of more intelligent equipment that assists in energy management. In addition, the use of a local database facilitates access to sensor data for a better study of equipment characteristics and implementation of machine learning.

The choice of humidity and power for the case study is due to the impacts on energy consumption and quality of food preservation, as excess humidity can generate ice accumulation and thus harm the motor's performance, in addition, the indication of high humidity may indicate problems related to the sealing of the doors and this may impact energy consumption. Nowadays, there are some refrigerators on the market with humidity sensors, but they do not have built-in machine learning. The analysis carried out highlighted that in addition to the number of times the refrigerator door is opened, the time it remains open is detrimental to the restoration of internal temperature and humidity, generating greater energy consumption.

Future work includes adapting the prototype in a smaller space, with the prototyping of PCB boards and 3D printing of a suitable case for the prototype, the use of OTA (Over-the-air) for a better software update, in addition, it is proposed to use the two ESP32 cores, one core for predicting humidity and the other for predicting motor activation. Another proposed improvement is comparative tests with inverter motors to evaluate the activation prediction, operating time, temperature, and humidity behaviour. Another investigation is about the time it takes for the temperature and humidity to return to their initial value every time the door is opened, in addition to the energy consumption spent during this period. Furthermore, relate the number of times the door is opened and the motor activation period.

## 9 Ethics statements

This work does not use any animal or human studies.

## CRediT authorship contribution statement

**Almir Neto:** Conceptualization, Data curation, Formal analysis, Investigation, Methodology, Software, Validation, Visualization, Writing – original draft, Writing – review & editing. **Luis Gomes:** Data curation, Formal analysis, Investigation, Methodology, Project administration, Software, Validation, Visualization, Writing – original draft, Writing – review & editing. **Zita Vale:** Conceptualization, Writing – review & editing, Visualization, Validation, Resources, Software, Supervision, Project administration, Methodology, Funding acquisition, Formal analysis.

## Declaration of competing interest

The authors declare that they have no known competing financial interests or personal relationships that could have appeared to influence the work reported in this paper.
